# Evaluation of a Density-Based Rapid Diagnostic Test for Sickle Cell Disease in a Clinical Setting in Zambia

**DOI:** 10.1371/journal.pone.0114540

**Published:** 2014-12-09

**Authors:** Ashok A. Kumar, Catherine Chunda-Liyoka, Jonathan W. Hennek, Hamakwa Mantina, S. Y. Ryan Lee, Matthew R. Patton, Pauline Sambo, Silvester Sinyangwe, Chipepo Kankasa, Chifumbe Chintu, Carlo Brugnara, Thomas P. Stossel, George M. Whitesides

**Affiliations:** 1 School of Engineering and Applied Sciences, Harvard University, Cambridge, Massachusetts, United States of America; 2 Department of Pediatrics, University Teaching Hospital, Lusaka, Zambia; 3 Department of Chemistry & Chemical Biology, Harvard University, Cambridge, Massachusetts, United States of America; 4 Department of Laboratory Medicine, Children's Hospital Boston, Boston, Massachusetts, United States of America; 5 Hematology Division, Brigham and Women's Hospital, Boston, Massachusetts, United States of America; 6 Wyss Institute for Biologically Inspired Engineering, Harvard University, Cambridge, Massachusetts, United States of America; Emory University/Georgia Insititute of Technology, United States of America

## Abstract

Although simple and low-cost interventions for sickle cell disease (SCD) exist in many developing countries, child mortality associated with SCD remains high, in part, because of the lack of access to diagnostic tests for SCD. A density-based test using aqueous multiphase systems (SCD-AMPS) is a candidate for a low-cost, point-of-care diagnostic for SCD. In this paper, the field evaluation of SCD-AMPS in a large (n = 505) case-control study in Zambia is described. Of the two variations of the SCD-AMPS used, the best system (SCD-AMPS-2) demonstrated a sensitivity of 86% (82–90%) and a specificity of 60% (53–67%). Subsequent analysis identified potential sources of false positives that include clotting, variation between batches of SCD-AMPS, and shipping conditions. Importantly, SCD-AMPS-2 was 84% (62–94%) sensitive in detecting SCD in children between 6 months and 1 year old. In addition to an evaluation of performance, an assessment of end-user operability was done with health workers in rural clinics in Zambia. These health workers rated the SCD-AMPS tests to be as simple to use as lateral flow tests for malaria and HIV.

## Introduction

Timely diagnosis of sickle cell disease (SCD) is essential for effective intervention. The difficulty of diagnosis of SCD in low-resource settings, however, means that over half of the more than 300,000 children born each year with this disorder die before five years of age [1], [2]. Measuring the mass density (g/cm^3^) of populations of erythrocytes provides a potential route to a low-cost, fieldable diagnostic for SCD. Density is a relevant biophysical characteristic differentiating red blood cells of a person with SCD from those cells of a person without the disease. The dehydration associated with sickling causes an increase in the density of an erythrocyte, from approximately 1.095 g/cm^3^ to over 1.120 g/cm^3^ [3], [4]. The dense, and often sickled, cells present in SCD are denser than the densest cells in the natural distribution of the density of normal erythrocytes.

We previously described the use of aqueous multiphase systems (AMPSs)—mixtures of polymers in water that form immiscible, liquid phases—to separate erythrocytes by density, and thus, to provide a rapid visual test for SCD [5]. Four characteristics make AMPSs suitable for density-based separations at the point-of-care: i) *Thermodynamic Stability*. Step-gradients can be made centrally and will reform after disturbances from transportation. ii) *Fine Resolution in Density*. The interface between the liquid phases of AMPSs provide a molecularly sharp step in density between phases with differences in density as low as 0.001 g/cm^3^ [6]. iii) *Scalability in Manufacturing*. Reproducible step gradients can be made by making large batches (>1 L) of an AMPS and then aliquoting smaller volumes (∼14 µL) of the AMPS into capillary tubes appropriate for separating microliters of blood obtained from a finger prick, and iv) *Low Cost*. Using commercially available polymers, the cost of reagents, packaging, and labor per test is ∼$0.50 [5].

Although previous testing of AMPS as a density-based diagnostic for sickle cell disease (SCD-AMPS) in a U.S. laboratory setting showed both sensitivity and specificity near or over 90% [5], implementing the test in point-of-care settings introduces several additional variables, such as accuracy in quality control, methods of packaging, conditions of storage and shipping, shelf-life, and variability in the skills and experience of end-users. These variables could reduce the sensitivity and specificity of a rapid test either via the tests themselves (e.g., dehydration could affect the assay) or the differences in operators (e.g., less experienced readers may have a lower diagnostic accuracy). Previous work on a paper-based test for liver function demonstrated that diagnostic accuracy could decrease from over 90% [7] to 84% [8] when tests were taken from a laboratory setting to a field setting. Evaluating tests in a field setting could illuminate issues in production, use, or interpretation that could be addressed with an improved design.

Testing SCD-AMPS on a larger population would also capture a greater diversity of samples. With a larger sample size, we could capture a larger range of conditions (e.g., severe anemia, α and β thalassemias) that could affect the performance of a density-based test. To understand how these issues might affect the SCD-AMPSs tests, we undertook a 505-subject case-control study at the University Teaching Hospital (UTH) in Lusaka, Zambia where sickle cell disease is prevalent.

We tested two prototypes, both of which demonstrated the ability to identify SCD with a diagnostic accuracy (defined as the total correct calls—true positives and true negatives—over the total number of samples tested) 69%. Based on the results of this study, we identified three key issues that could be addressed in continuing work to improve the performance of future prototypes: 1) variability in the density of the bottom phase between batches, 2) conditions of shipping and storage, and 3) clotting of blood samples. We also performed surveys of health workers in two rural health centers in a part of Zambia estimated to have a high prevalence of SCD [9]. The surveys captured current knowledge of the disease, experience with other rapid tests, and feedback on the design of the proposed rapid test for SCD.

## Experimental Design

### Rapid Test Design

**Figure 1:**
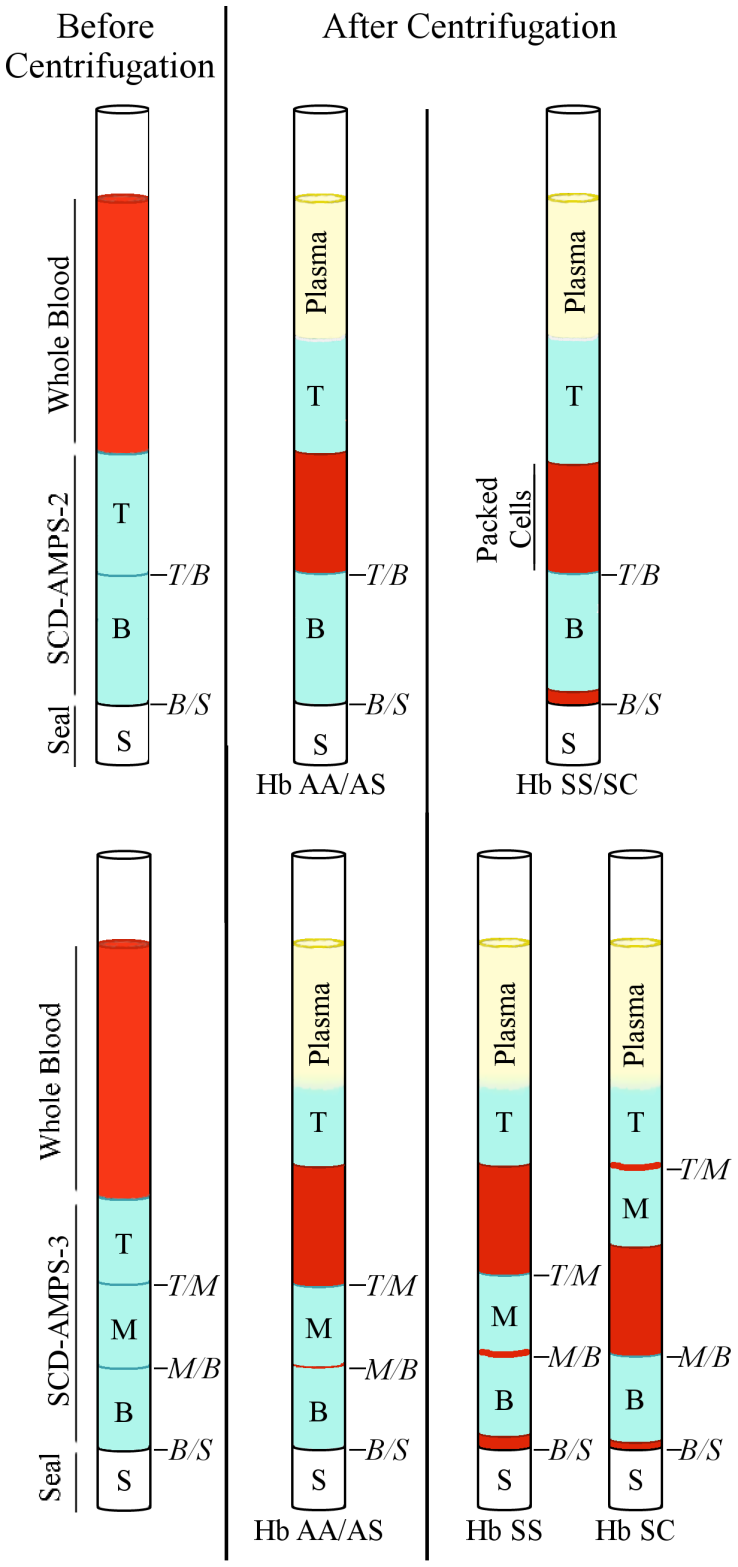
Schematic of the density-based tests to identify SCD. Both versions of the SCD-AMPS are designed to separate dense red blood cells present in SCD from whole blood. Blood passes through the phases—top (T) and bottom (B) for SCD-AMPS-2 and top (T), middle (M), and bottom (B) for SCD-AMPS-3—upon centrifugation. If sickled cells are present, they collect at the interface between the bottom phase and the seal (*B/S*), and provide a visual readout for the presence of SCD. In SCD-AMPS-3, the additional phase allows the discrimination of Hb SS from Hb SC by evaluating the distribution of red cells at the upper interfaces (between the top and middle phases (*T/M*) and the middle and bottom phases (*M/B*).

We evaluated two different density-based tests: a system with two phases, SCD-AMPS-2, and a system with three phases, SCD-AMPS-3 (**Fig. 1**). Sickling is concomitant with the dehydration of red blood cells. The resulting deformed cells have a marked increase in density. By tuning the density of the bottom phase of an AMPS to retain cells with normal densities and only allow very dense cells (Δρ>1.120 g/cm^3^) to pass to the bottom of the AMPS, we can provide a visual test for dense cells that may correlate with the presence of SCD [5]; red blood cells at the bottom interface (i.e., between the bottom phase and the seal of the container) create a red layer. The design of the bottom phase is common to both SCD-AMPS-2 and SCD-AMPS-3. The top phase in both systems separates plasma from cells and ensures that cells stack at well-defined interfaces rather than a diffusive liquid/liquid boundary. The third phase in SCD-AMPS-3 provides an additional phase and interface to separate cells in the lower density range of erythrocytes.

Although SCD-AMPS-2 allows for a simpler interpretation, SCD-AMPS-3 provides a richer set of information about the distribution of densities of erythrocytes; this information can help distinguish between the two main genotypes of SCD: Hb SS and Hb SC [5]. We evaluated both tests in the study to see if the simplicity of the two-phase test conferred a distinguishable advantage over the additional information available in the three-phase tests, in an actual clinical setting.

We used the design of the rapid test described previously [5] with some additional modifications to enable rapid assembly of the tests, and to improve durability for shipping and storage (**S1 Figure**). A mixture of 7.0% (w/v) poly(ethylene glycol) (PEG) with a molecular weight (MW) of ∼20 kD, 10.3% (w/v) Ficoll with a MW of ∼400 kD, and 9.1% (w/v) Nycodenz formed SCD-AMPS-2 (ρ_top_ = 1.078 g/cm^3^ and ρ_bot_ = 1.129 g/cm^3^). Similarly, a mixture of 3% (w/v) PEG with a MW of ∼20 kD, 10% (w/v) dextran with a MW of ∼500 kD, 5% (w/v) polymer of partially hydrolyzed poly(vinyl acetate) (containing 75% -OH and 25% -OCOCH_3_ groups) with a MW of ∼3 kD, and 8.7% (w/v) Nycodenz, formed SCD-AMPS-3 (ρ_top_ = 1.077 g/cm^3^, ρ_mid_ = 1.108 g/cm^3^, and ρ_bot_ = 1.120 g/cm^3^). We buffered the systems, and used NaOH and HCl to adjust the pH of the solutions to 7.40±0.02 and NaCl to adjust the osmolality to 295±15 mOsm/kg (**S1 Text**).

**S2 Figure** outlines the process to perform a rapid test for an end-user. The self-forming step-gradient in density allows the end-user to use the AMPS out of a packet without needing to mix reagents or pipette solutions. A drop of whole blood wicks directly into the capillary; no further handling of blood is necessary. Preparative steps common to other techniques, such as lysis or exposure to reagents to deoxygenate hemoglobin [10], [11], are not needed. These characteristics of the test reduce risks for biohazards and error by users. The polycarbonate capillary tubes that house the rapid test are preloaded with AMPS and sealed on one end with white, vinyl-based putty (Critoseal, Leica). The white seal provides an effective background to contrast with the red cells. A volume of 5.0±0.2 µL of blood enters the rapid test via capillary action; the volume is limited by the pre-punched hole in the side of the capillary. We used a 3D printed mold and a pushpin to make repeatable holes; this standardization allowed us to load a reproducible volume of blood with a coefficient of variance (CV) of 4% (**S1 Text**). Sliding a silicone sleeve over the hole prevents the blood from leaking, and centrifugation accelerates the density-based separation of red blood cells across the steps in density of the AMPS. After 10 minutes of centrifugation at 13,700 *g*, evaluating the interfaces of AMPS for the visible presence of red cells provides a means to identify SCD and, in the case of SCD-AMPS-3, to distinguish between the two main genotypes of SCD (**Fig. 1**) [5].

### Development of Methods to Pack, Store, and Ship SCD-AMPS Tests

We performed a series of accelerated storage tests using various packaging materials and methods (**S1 Text**). Individual capillaries were sealed on each end, and the hole in the side of the tube was covered with the silicone sleeve before packaging. We found that foil-lined pouches to which we had added 4 mL of water, and then sealed with an impulse sealer, minimized evaporation of the water from the AMPS in the tubes. To minimize variables for this clinical trial, we refrigerated the rapid tests after packaging at 4–8°C and shipped them on ice to Zambia. On arrival in Zambia, the staff at UTH stored the samples at 4°C. We brought the tests to room temperature on the day of use. Each batch of packaged tests was used within two months of the packaging date. Future work will define the shelf life and stability of the tests during storage at ambient temperatures.

### Characteristics of Population for the Clinical Study

The initial study in Zambia included 505 children that were seen as out-patients or in-patients between June and December, 2013, in the Department of Paediatrics and Child Health and the hematology unit for SCD patients at UTH. We used the broad inclusion and exclusion criteria found in **Table 1**, with further criteria for specific subsets of the study population: Subset 1) children fitting the inclusion criteria with the additional inclusion criteria of being over one year old and confirmed as SCD positive, Subset 2) children fitting the inclusion criteria above with the additional inclusion criterion of not having SCD, Subset 3) children fitting the inclusion criteria with the additional inclusion criteria of being below one year old and confirmed as SCD positive, and Subset 4) children fitting inclusion criteria with the exception of the first exclusion criteria who were over one year old, confirmed as SCD positive, and had undergone a sickling crisis within the last 48 hours.

**Table 1:** Inclusion and Exclusion Criteria for Study.

Inclusion Criteria	Exclusion Criteria
▪ Children aged 6 months up to, but not including 18 years	▪ Children who have had a sickling crisis one month prior to the blood draw (except for the subset specified below)
▪ Children with clinical indication for a blood draw	▪ Children who have been treated with hydroxyurea in the last four months
▪ Children whose parents give a written informed consent to be part of the study	▪ Children who have received a transfusion in the last four months
▪ Children whose parent consent to have blood draw for clinical purposes and for the study	

The first two subsets were recruited to achieve a population with roughly 50% SCD positive participants. These participants provide the main population of interest in the study. The last two subsets were of interest to test potential confounding factors for a field diagnostic. Specifically, before achieving one year of age, infants may still have a large proportion of fetal hemoglobin, Hb F, in their blood. This hemoglobin interferes with sickling and could reduce the percent of the cells present that are dense, and reduce the sensitivity of a density-based assay for this subset. An evaluation of this subset allowed us to determine whether there was a difference between the predictive value of the SCD-AMPS test for children below one year of age and children above one year of age. Similarly, participants who have SCD and have recently experienced a sickle crisis may have cleared all dense cells in their blood [12], although that finding has not been confirmed. **Table 2** details the the final populations used in the study.

**Table 2:** Basic Characteristics of the Study Population.

Population	Total		
	Subjects	Male	Female
**Positive (Hb SS)**	322	166	156
**≥** ***1 yr, non-crisis***	*270*	*144*	*126*
***<1 yr, non-crisis***	*19*	*4*	*15*
***>1 yr, crisis***	*33*	*18*	*15*
**Negative**	183	91	92
*** Hb AA***	*136*	*65*	*71*
*** Hb AS***	*47*	*26*	*21*
**TOTAL**	**505**	**257**	**248**

The 505 subjects included in the study were a subset of 767 total subjects that were recruited who fit the inclusion criteria. Of these 767 subjects, 43 were excluded because their blood sample clotted or was of insufficient volume to perform all the tests. A batch of SCD-AMPS tests sent to Zambia was made with an incorrect buffer concentration. When this error was realized, the study staff were informed to not use these tests but 60 subjects had already been tested with them and were excluded from the main study. Of the 664 subjects remaining, 159 were not tested on SCD-AMPS until after 48 hours (outside the inclusion limit). Eight of these were not tested until after 72 hours. The final number of subjects that had usable samples run on valid tests within the proper timeframe was 505 (**S3 Figure**).

### Collection of Samples

In both in-patient and out-patient clinics, we recruited participants who were having blood drawn for clinically indicated reasons. An additional vial of blood was collected for the study after consenting the participants. This blood was aliquotted into two different samples. One sample was sent to a clinical laboratory at UTH to perform the SCD-AMPS tests and the other sample was sent to a separate laboratory at UTH to perform evaluation of the sample using standard methods.

### Evaluation of Samples by Standard Methods

We chose to evaluate the SCD-AMPS tests at UTH, a tertiary hospital, rather than lower level clinics because of the need to compare results to standard methods. UTH has well-established clinical laboratories capable of performing hemoglobin electrophoresis (HE) and complete blood counts (CBCs), and they receive regular shipments of reagents from vendors. Both HE and CBCs were performed on all the samples. The rapid tests and confirmatory tests occurred at different laboratories and were performed by separate personnel to ensure proper blinding of the results. During the pilot phase of the study, we established the maximum time between sample collection and testing for each type of analysis, as well as other criteria that we used to bin samples as unusable (e.g. visible clots forming and insufficient volume of blood to run all tests) (**S1 Table**). These criteria were used to exclude samples or tests. Results from HE and CBC allowed us to classify subjects as SCD, sickle cell trait, or non-SCD based on detecting the presence and quantity of Hb S as well as evaluation of the red blood cell indices (**S1 Text**).

### Visual Inspection and Validation

**Figure 2:**
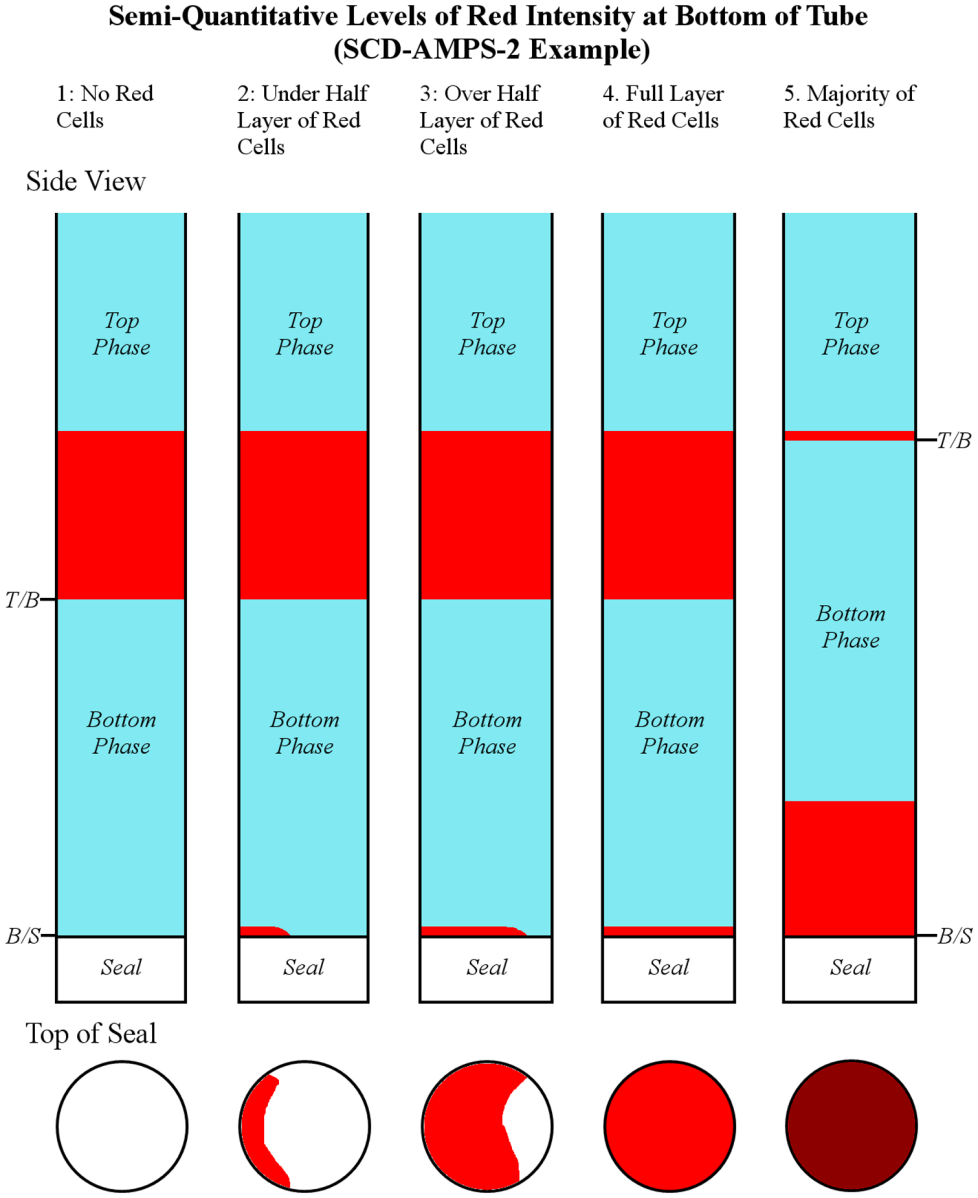
Schematic representation of the five levels of evaluation of the bottom of an SCD-AMPS test. Levels 1 through 5 correspond to increasing levels of accumulation of red cells at the interface between the bottom phase and the seal (*B/S*) compared to the accumulation of red cells at the interface between the top and bottom phase (*T/B*).

After a one-day period of training, readers (nurses and laboratory technicians at the hospital) were instructed to evaluate each test by classifying the amount of red color that was visible at each interface into bins: 1) no red cells detectable, 2) less than half a layer of red cells, 3) over half a layer of red cells, 4) full layer of red cells, or 5) majority of red cells (**Fig. 2**).

We chose to use these five classifications rather than a simple binary reading in order to understand how different cut-offs could affect test performance. For the previous study at Harvard University, we had used a full layer of red or more (i.e. classification 4 or higher) at the bottom interface as the criterion for labeling results as positive or negative, and, in the case of SCD-AMPS-3, the identification of the majority of cells at the lower liquid interface (i.e. classification of 5) as indication of the presence of either Hb SC or Hb CC [5]. Although the five classifications added complexity to interpretation, they allowed a greater resolution of the potential differences in the densities of cells.

### Field visits to obtain feedback from end-users

We performed demonstrations of the rapid test, and took suggestions on the use of the test, from the clinical staff at two rural health centers (RHCs) in the Luwingu District of Northern Province, Luena RHC and Ipusukilo RHC. The Northern Province is estimated to have a high prevalence of SCD [9]. We selected the two centers in the Northern Province for their remote location with no access to grid electricity or paved roads. At each site, over the course of two days, study staff members provided an information session about sickle cell disease and management for the clinical staff, community health workers, and the broader community, as well as an information session and demonstration of the SCD-AMPS-2 rapid test. Following these sessions, a survey of the clinical staff members (who included nurses and clinical officers) and community health workers assessed their familiarity with other rapid tests, and their thoughts on the SCD-AMPS-2 rapid test. A member of the study team from Harvard performed the demonstration of the test using his own blood in accordance with an IRB-approved protocol.

## Results and Discussion

### The Packaging Method Prevented the Evaporation of Samples

All rapid tests were evaluated visually for irregularities after a short centrifugation (2 minutes at 13,700 *g*) before use (**S2 Figure**), and the result of this inspection was logged for the vast majority of the tests used (n = 624). We found two modes of failure of the rapid test after packaging, shipping, and storing. In 3.8% of the tests, the plug from the rubber cap snapped upon removal from the tube, leaving the capillary plugged and unusable. In 1.5% of the tests, we observed incorrect liquid levels; 1.0% were under-filled and 0.5% were over-filled. Water could enter or exit the test through three faults: 1) incomplete sealing with the vinyl putty and glue at the bottom of the test, 2) improper capping of the top of the test with the rubber stopper, or 3) incomplete coverage of the hole in the side of the capillary by the silicone sleeve. Notably, all tests formed the correct number of phases as verified by visual inspection. The total failure rate for the packaging, shipping, and storage of the rapid tests was 5.3%. Visual inspection without the short centrifugation may be sufficient since phases always formed properly in such a large sample size, but such a change in procedure will need to be validated. Tubes that failed these visual inspections were discarded and not used for testing blood samples.

### Performance of SCD-AMPS

**Figure 3:**
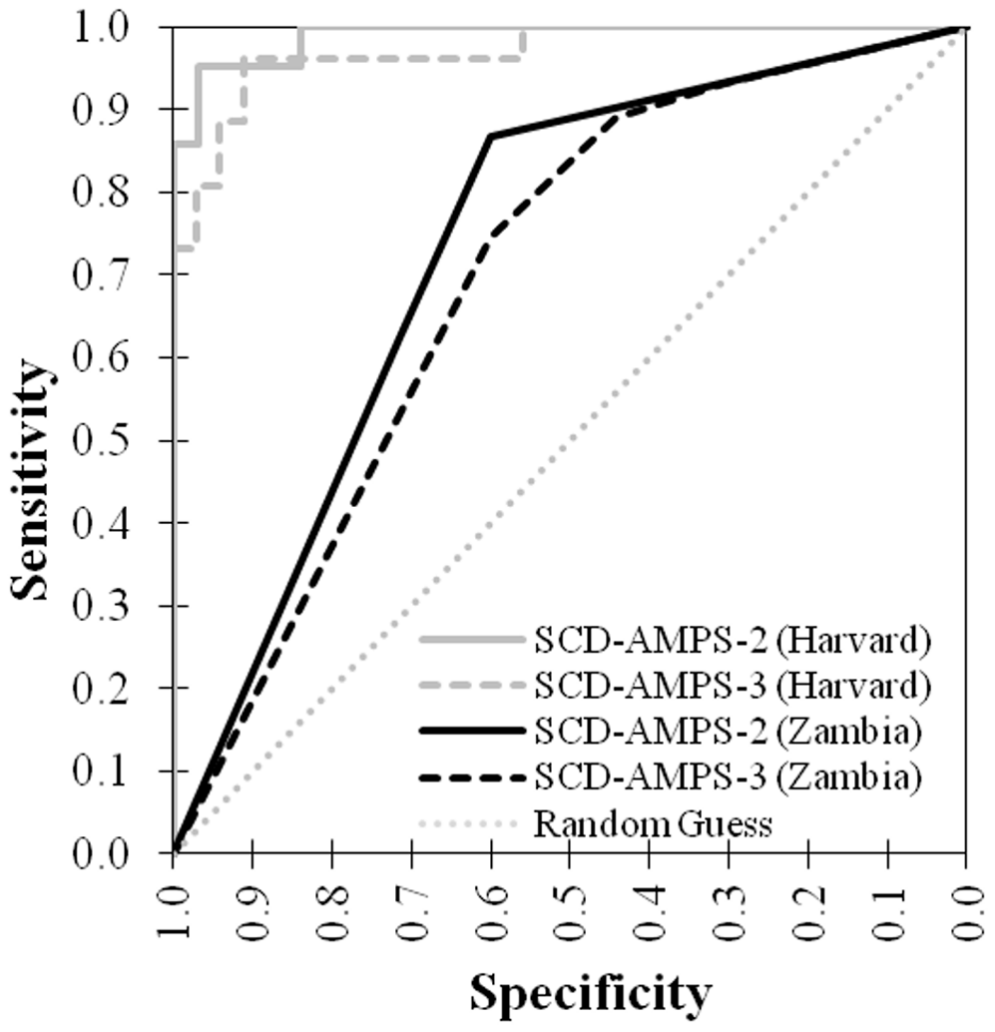
Receiver Operating Characteristic (ROC) curves of SCD-AMPS-2 and SCD-AMPS-3 including all data from Zambia shows fair discriminative ability. The amount of red cells below the bottom phase of each test was evaluated by eye and classified on a five point scale. Both tests showed an ability to discriminate sickle cell disease from non-disease. In general, the specificity was lower than the sensitivity of the tests.

We calculated the sensitivity and specificity of SCD-AMPS-2 and SCD-AMPS-3 using each of the five classifications for the level of red cells at the bottom interface (i.e., the interface between the lower phase and the seal) (**Table 3**). We constructed receiver operating characteristic curves using these calculations (**Fig. 3**). The area under the curve (AUC) for SCD-AMPS-2 was 0.73 and for SCD-AMPS-3 was 0.70. This indicates an ability to discriminate sickle cell disease, but is lower than previous estimates of performance (AUC>0.95) found during initial validation of these systems in a laboratory setting [5].

**Table 3:** Tabulation of Results of SCD-AMPS Tests Compared to Reference Test Results by Hemoglobin Electrophoresis.

*SCD-AMPS-2*	Hemoglobin Electrophoresis			
	SCD Negative		SCD Positive	
Red Level at Bottom	AA	AS	SS	AS*
(1) None	82	28	37	6
(2) Under half layer	5	1	21	2
(3) Over half layer	10	5	55	0
(4) Full layer	39	13	196	4
(5) Majority of red	0	0	1	0
Excluded	140	17	58	4

*SCD-AMPS-3*	Hemoglobin Electrophoresis			
	SCD Negative		SCD Positive	
Red Level at Bottom	AA	AS	SS	AS*
(1) None	39	17	18	4
(2) Under half layer	21	4	12	1
(3) Over half layer	19	10	44	3
(4) Full layer	57	16	236	4
(5) Majority of red	0	0	0	0
Excluded	140	17	58	4

*Samples found to have>50% Hb S but non-zero levels of Hb A, potentially a result of Hb S with β-thalassemia or a transfused Hb SS subject.

We used the threshold at which the product of the sensitivity and specificity was highest as the evaluation threshold for each of the two SCD-AMPS. For SCD-AMPS-3, this level was “less than half a red layer” at the bottom of the tube or higher to be considered positive (Level 2). For SCD-AMPS-2, the cutoff was a “full layer of red” at the bottom of the tube or higher (Level 4). Using these thresholds, visual readings of SCD-AMPS-2 provided a sensitivity of 87% with a 95% confidence interval (CI) of 82–90%, a specificity of 60% (CI 53–67%), and an overall diagnostic accuracy of 77% (CI 73–81%); and SCD-AMPS-3 had a sensitivity of 75% (CI 70–79%), a specificity of 60% (CI 53–67%), and an overall diagnostic accuracy of 69% (CI 65–73%).

In 2013, the National Institute of Health (NIH) in the U.S. put out a request for proposals to develop new point-of-care diagnostics for SCD. They suggested that new tests should have a sensitivity of over 60% and a diagnostic accuracy of over 90% (RFA-HL-14-010). In children over 6 months old in this study, SCD-AMPS demonstrate sensitivity well above the recommendation, but specificity will need to be improved to achieve the suggested diagnostic accuracy. Furthermore, evaluation on children below 6 months of age is still needed to assess whether a density-based test could be used on newborns.

Part of the reduced specificity could be attributed to the bias of the readers at UTH to read samples with a higher level of redness compared to the expert reader (**S1 Text**). The use of a cell phone camera or portable scanner to analyze the rapid tests would eliminate the subjectivity associated with visual inspection.

The trial in Zambia introduced several other variables that may have played a role in lowering the performance as compared to results obtained with clinical samples tested previously at Harvard University [5], and are potential areas for improvement for development of this point-of-care diagnostic. We analyzed several of these variables and concluded that three of them were the most probable causes of the reduced performance: 1) storage and shipping conditions, 2) variability in the batches of AMPS (**S4 Figure**, **S2 Table**)—particularly, variations in the density of the bottom phase between batches—and 3) clotting (**S1 Text**).

Five batches of SCD-AMPS tests were made at Harvard and shipped to UTH during the course of the study (a sixth batch was also used, but was incorrectly formulated and, thus excluded). Comparing the diagnostic accuracy of each batch with Fisher's exact test shows that performance of both SCD-AMPS varied significantly (p-value <0.001) between batches. Slight variations in the density of the bottom phase of each batch may have influenced the performance of the tests; this issue could be addressed by implementing tighter quality control at the site of production and by developing density standard beads for the tests to provide experimental controls. All batches were shipped with ice packs via FedEx, generally arriving at UTH five days after dispatch from Harvard. The third batch, however, was delayed in transit due to a fire at the Nairobi International Airport that disrupted travel and shipping throughout sub-Saharan Africa. This batch took ten days to arrive at UTH. The SCD-AMPS-2 from Batch 3 had the second lowest diagnostic accuracy of the batches, and the SCD-AMPS-3 tests from that batch had the lowest diagnostic accuracy of all batches (**S4 Figure**). Without data loggers for temperature and humidity, we can only speculate about whether the additional transit time resulted in an extreme condition, and, thus, we have included the results of tests from the third batch in the overall analysis. Notably, performance improves when these results are excluded; SCD-AMPS-2 improves to a sensitivity of 90% (85–93%) and a specificity of 64% (55–72%), and SCD-AMPS-3 improves to a sensitivity of 79% (73–83%) and a specificity of 70% (62–78%).

Variability in the total blood drawn from each subject may have led to samples receiving different concentrations of ethylenediaminetetraacetic acid (EDTA). We found that both under treatment and over treatment with EDTA could lead to false positives as a result of clotting or dehydration from high levels of EDTA (**S5 Figure**). The heparin coating on the capillary tubes had a negligible effect on clotting. The eventual use of SCD-AMPS directly from finger prick samples necessitates future work to incorporate effective anti-coagulants into the blood collection aspect of the SCD-AMPS tests.

**Figure 4:**
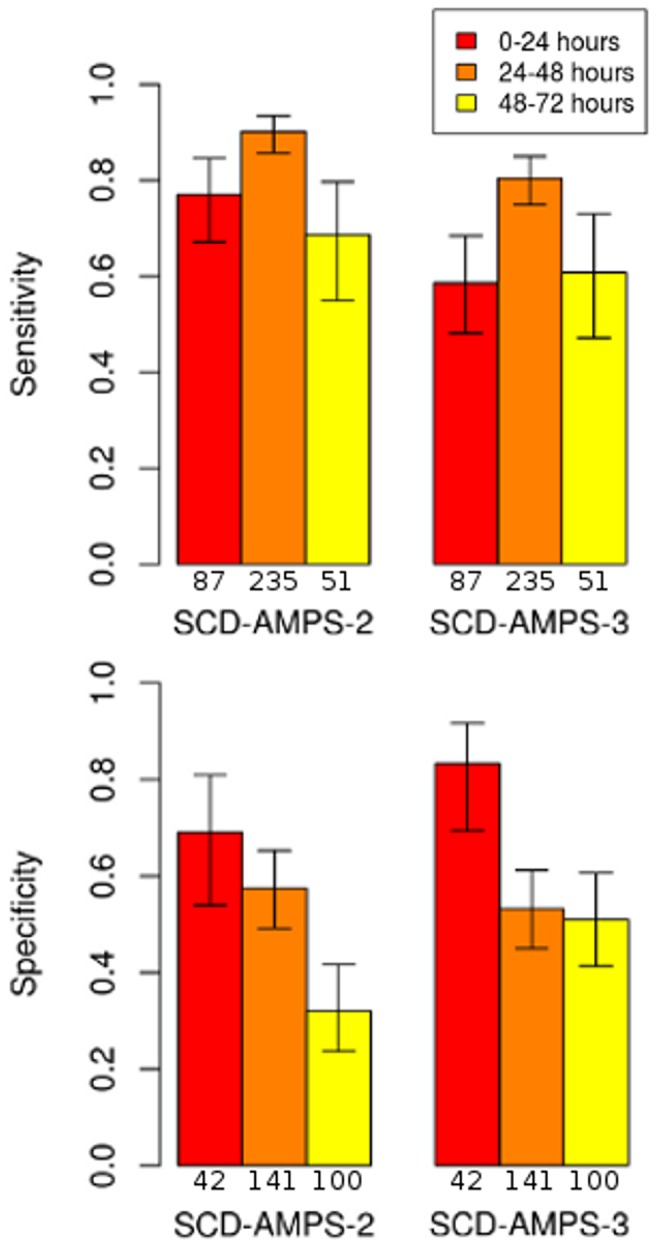
The sensitivity and specificity of SCD-AMPS as a function of the amount of time between collecting samples and running tests. The specificity shows a decline over each 24 hour increment, with a significant decline over 48 hours (p-value <0.0005). The sensitivity increased between the first and second time interval, but then decreased between the second and third interval (p-values <0.01). The sample size used for each time interval is provided below each bar.

Based on our previous work [5], we set 48 hours after blood was drawn as a cutoff for inclusion in the study. To test the effect of time after collection to running tests, we also analyzed results from samples run after 48 hours. Binning test results based on whether they were run in the first, second, or third 24 hour time period after collection demonstrated a clear decline in specificity over time (**Fig. 4**). Both SCD-AMPS-2 and SCD-AMPS-3 showed significant (p- value  = 7.5×10^−5^ and 3.1×10^−4^) decline between samples run within 24 hours and those run after 48 hours. In both systems, sensitivity increased between the first two time intervals (p-value  = 0.0031 and 1.5×10^−4^) and decreased between the last two time intervals (p-value  = 2.0×10^−4^ and 0.0053) (**Fig. 4**). These results indicate that storage of the blood samples has an impact on the effectiveness of the tests. Storing erythrocytes in refrigeration can cause morphological changes that may affect density [13]. Testing on samples collected directly from a finger prick and run immediately could provide a more accurate prediction of performance of SCD-AMPS.

The capillaries used to fabricate the tests were coated in sodium heparin and the AMPS themselves contained EDTA. The combination of these anticoagulants with blood that was already treated with anticoagulant may have led to increased dehydration of erythrocytes and caused some false positives [14].

Future work on the development of SCD-AMPS as a rapid test should focus on quality control to reduce batch to batch variations in density, evaluation of shelf-life and stability in various shipping conditions, optimization of the type and concentration of anticoagulant in the devices, the creation of reliable positive and negative controls, and assessing effectiveness when used on fresh blood samples from finger pricks rather than venous blood that has been stored at 4°C.

### Performance on Subpopulations

We evaluated several subpopulations of interest to determine whether the SCD-AMPS tests would be suitable for general screening (**Table 4**). One concern with a diagnostic test based on the dense cells present in SCD is that other concurrent conditions may lead to a decrease in the number of dense cells in circulation and compromise the sensitivity of the diagnostic.

**Table 4:** Sensitivity of SCD-AMPS tests on specific subpopulations.

	SCD Subjects	*SCD-AMPS-2*			*SCD-AMPS-3*		
Population		Sensitivity	CI	p-val.^a^	Sensitivity	CI	p-val.^a^
*All*	*322*	*87%*	*(82,90)*	*—*	*75%*	*(70,79)*	*—*
6–12 months	19	84%	(62,94)	7.3×10^−1^	53%	(31,73)	3.1×10^−2^
Recent Crisis^b^	33	94%	(80,98)	2.8×10^−1^	88%	(73,95)	8.9×10^−2^
Hb F>20%	36	58%	(42,70)	5.4×10^−6^	39%	(25,55)	1.5×10^−6^
Micro. Hypo.^c^	89	80%	(70,87)	2.9×10^−2^	62%	(51,71)	1.6×10^−3^
Sev. Anem.^d^	112	93%	(87,96)	1.6×10^−2^	86%	(78,91)	7.4×10^−4^
Hb S>50%, Hb A>0%^e^	12	50%	(25,75)	2.0×10^−3^	33%	(14,61)	2.6×10^−3^

a p-value from a two-sided version of Fisher's exact test comparing samples from a subpopulation to the total population excluding the subpopulation.

b Self-reported to have experienced body pain or vaso-occlusive crisis within previous 48 hours.

c Micro. Hypo. – Microcytic & hypochromic anemia defined as patients with a mean corpuscular volume below 80 fL, a mean corpuscular hemoglobin below 26 pg/cell and a hemoglobin concentration below 13.5 g/dL for males or below 21.0 g/dL for females.

d Sev. Anem. – Severe anemia defined as a hemoglobin concentration below 8 g/dL for those over 5 years old, and 7 g/dL for those less than 5 years old.

e Subjects that may either have Hb Sβ+ or may be Hb SS with a recent transfusion

The high levels of Hb F in very young children (below 1 year old) has a protective effect in SCD [15], [16]. To test whether this effect would reduce sensitivity, we evaluated a subpopulation of subjects with SCD that were between 6 months and 1 year of age, as well as a subpopulation of subjects with SCD that had Hb F>20%. High levels of Hb F resulted in significantly decreased sensitivity in both SCD-AMPS-2 and SCD-AMPS-3 (p-value <0.001). Interestingly, SCD-AMPS-2 showed a sensitivity for those between 6 months and 1 year of age (84%) similar to that in the general population (87%). Sensitivity of SCD-AMPS-2 for this subpopulation compared to sensitivity in children over 1 year old was not significantly different (p-value  = 0.73).

The occurrence of a recent vaso-occlusive crisis has been reported to reduce the percent of dense cells present in SCD [12]; we, therefore, tested a subpopulation of patients with SCD who self-reported to have experienced a crisis in the last 48 hours (n = 33). In both SCD-AMPS-2 and SCD-AMPS-3, the sensitivity was similar or higher for this subset compared to subjects who had not had a recent crisis.

Severe anemia (defined as a hemoglobin concentration below 8 g/dL for children over 5 years of age and 7 g/dL for children under 5 years old)[17] is common among patients with SCD. In both SCD-AMPS systems, however, those with both SCD and severe anemia were detected with high sensitivity (>85%). Microcytic, hypochromic anemia—the condition of having a mean corpuscular volume below 80 fL, mean corpuscular hemoglobin below 26 pg/cell and hemoglobin concentration below 13.5 g/dL for males and 21.0 g/dL for females—could indicate the presence of iron deficiency anemia or α-thalassemia [18], [19]. For both versions of SCD-AMPS, the sensitivity on patients with SCD that had microcytic, hypochromic anemia was lower than on those without it (p-value <0.05) (**Table 4**). The performance of density-based tests may be affected by these conditions, which can decrease the density of erythrocytes. Despite reduced performance, SCD-AMPS-2 still attained 80% (70–87%) sensitivity on those with SCD and microcytic, hypochromic anemia.

12 subjects were found to have Hb S>50% but also to have Hb A>0% (**Table 3**). This finding could indicate the subjects had β+ thalassemia and an Hb S gene. Alternatively, these subjects may have been Hb SS patients who had received a blood transfusion from someone with Hb AA. Although the study was designed to exclude recently transfused individuals, incomplete medical records meant that study staff had to rely on the responses of participants and guardians to surveys on whether a transfusion had been received in the past four months and may have had some inaccuracies. In these 12 subjects, sensitivity of the test was significantly decreased (p-value <0.01) (**Table 4**).

Without the ability to perform genetic testing at the site of the study, we were unable to confirm the concurrent presence of either α or β thalassemias. Concurrent thalassemias could affect the number of dense cells in SCD [3], [20]. The reduced performance of SCD-AMPS in samples with microcytic, hypochromic anemia and in subjects with Hb S>50% but Hb A>0% indicates that the effect of both α and β thalassemias could be important. Larger sample sizes and confirmatory testing will be required to assess the full impact of these conditions on diagnostic accuracy.

On both tests, specificity was not significantly different (p-value  = 1.0 for SCD-AMPS-2, and p-value  = 0.39 for SCD-AMPS-3) for subjects with Hb AA and Hb AS; sickle cell trait was not a major factor in creating false positives (**S3 Table**). This characteristic sets density-based tests apart from solubility tests like Sickledex [21].

Although the reduction in performance due to high Hb F merits some caution, the high sensitivity for children between 6 months to 1 year of age, patients with a recent vaso-occlusive crisis, and those with severe anemia suggests that the SCD-AMPS-2 test should be useful as a screening or diagnostic tool for children as young as 6 months old. Similarly, SCD-AMPS-3 should be beneficial if used on children over 1 year of age.

### SCD-AMPS-2 Outperformed SCD-AMPS-3

One of the explicit goals of this trial was to establish whether the two different tests (SCD-AMPS-2 and SCD-AMPS-3) had differences in performance in a large clinical test. The threshold that produced the best combination of sensitivity and specificity for each test was different. SCD-AMPS-2 performed best with the criterion that the bottom of the tube had a full layer of red, whereas SCD-AMPS-3 was best when the more permissive minimum of any detectable amount of red (including less than half a layer of red). Using these thresholds for each test, we compared performance. SCD-AMPS-2 had a significantly (p-value <0.01) higher diagnostic accuracy (77% with C.I. 73–81%) than SCD-AMPS-3 (69% with C.I. 65–73%). An alternative to diagnostic accuracy is to use the diagnostic odds ratio (DOR), defined as (true positives × true negatives)/(false positives × false negatives). This ratio provides a measure of performance that is not as affected by imbalances in recruitment of positives and negatives as diagnostic accuracy. The logarithm of the DOR is normally distributed and allows us to calculate a standard deviation (S.D.). With a log DOR of 2.3 (S.D.  = 0.2), SCD-AMPS-2 again outperformed SCD-AMPS-3, which had a log DOR of 1.5 (S.D.  = 0.2).

One of the potential benefits of SCD-AMPS-3 over SCD-AMPS-2 is the potential ability of the three-phase system to distinguish between the Hb SC and Hb SS variant of SCD [5]. In this study, however, none of the patients had the Hb SC genotype, and thus, we cannot conclude whether SCD-AMPS-3 can identify Hb SC from this study. Hemoglobin C is more common in West Africa [22], and a future study in that area could elucidate the ability of the test to distinguish this subtype. In practical use, the choice of method could be selected based on known epidemiological information about a specific region; SCD-AMPS-2, for example, might be best in Zambia, while SCD-AMPS-3 may be more useful in Mali. Individuals with Hb SS and Hb SC genotypes, furthermore, have different clinical presentations that clinicians could take into consideration with the results of testing.

Despite the inability to validate the ability of SCD-AMPS-3 to identify Hb SC on positive samples, we could test whether SCD-AMPS-3 would incorrectly identify an Hb SS sample as Hb SC. Of the 505 subjects tested, only three had an SCD-AMPS-3 test with the majority of cells at the lower liquid interface (as opposed to the upper liquid interface). One of these subjects also had a full layer of red color at the bottom of the three-phase system and the HE results confirmed the person as Hb SS. This result would correspond to a misidentification of Hb SS as Hb SC in <1% of cases. The other two tests with the majority of cells at the lower liquid interface came from one person with Hb AS and one with Hb AA. In both cases, no red color was detected at the bottom of SCD-AMPS-3, and, therefore, both samples were correctly classified as not having SCD.

Even though the two SCD-AMPS tests demonstrated similar performance in early validations studies at Harvard University [5], the trial in Zambia demonstrates that SCD-AMPS-2 is more robust as a diagnostic test than SCD-AMPS-3. Apart from the difference in the number of phases of these two systems, a potential difference that could account for the variation in performance is the density of the bottom phases of the AMPSs. SCD-AMPS-2 was designed to have a bottom phase with a density of 1.129 g/cm^3^ while SCD-AMPS-3 had a less dense bottom phase with a density of 1.120 g/cm^3^. A reformulation of a three phase system with a bottom phase with a higher density than the current SCD-AMPS-3 may provide a performance that is more similar to SCD-AMPS-2.

### Capabilities of Rural Health Centers

A point-of-care diagnostic provides benefit when it is coupled to effective interventions. Simple interventions (e.g., pneumococcal vaccine (PCV) and prophylactic antibiotics) for sickle cell disease exist, and we sought to assess whether rural health centers in Zambia had the capabilities to perform an SCD-AMPS test and provide appropriate interventions.

Working with the U.S. Peace Corps in Zambia, we identified two Rural Health Centers (RHCs)—Luena RHC and Ipusukilo RHC—in Northern Province to carry out a demonstration of the rapid test and an assessment of capabilities to care for patients with SCD. Our survey indicated that the clinical staff at both RHCs was well acquainted with rapid tests for malaria, HIV, and syphilis and had a supply of these tests. Neither clinic had a microscope or capabilities to diagnose SCD with any of the currently available methods. Although both clinics lacked the infrastructure to perform transfusions and did not have morphine, they did have the ability to provide several other interventions that have been shown to reduce mortality and ease the symptoms of sickle cell disease, including folic acid supplements, intravenous fluids, antibiotics, antimalarials, and PCV (**S4 Table**) [23]–[25].

### Feedback on SCD-AMPS from End-users

**Figure 5:**
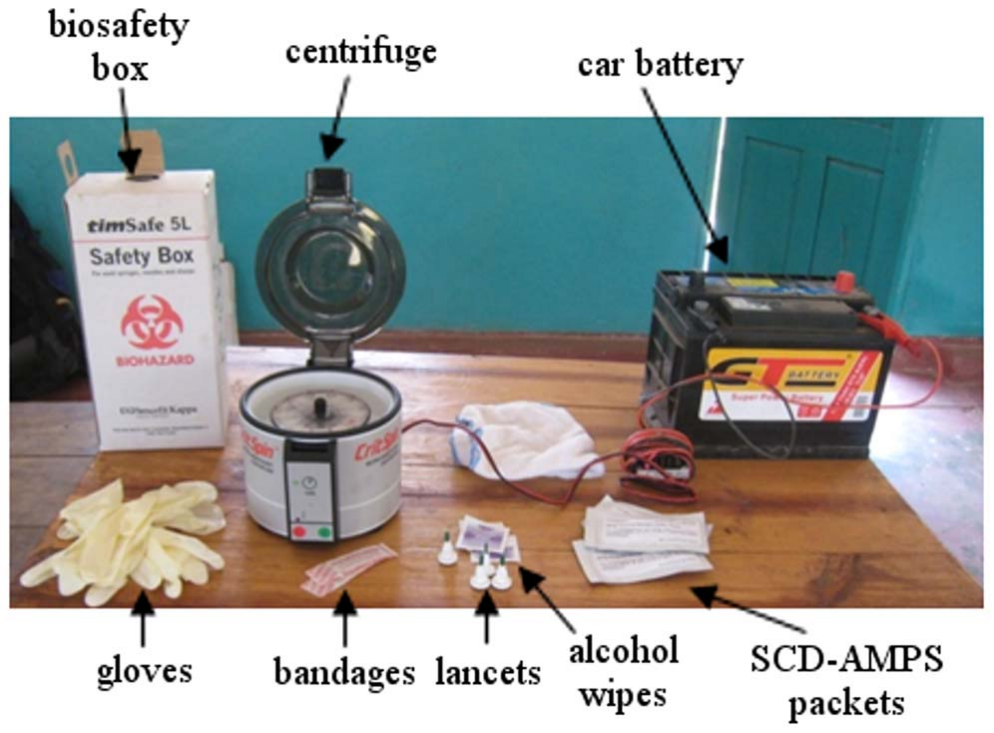
Equipment for the SCD-AMPS rapid test. All the equipment necessary to run the rapid test in a rural clinic fits inside a backpack and were evaluated at rural health centers in Zambia.

The SCD-AMPS tests were designed to use minimal equipment in order to be accessible to low-level clinics. Apart from the SCD-AMPS tests themselves, the only other equipment necessary to perform a test is a microhematocrit centrifuge. We used a centrifuge (CritSpin, Iris Sample Processing) powered by a car battery with a DC-to-DC converter. The tests, supplies to perform a finger-prick safely, the centrifuge, and the battery all fit into a backpack (**Fig. 5**) and were transported from Lusaka to the RHCs by bus, shared taxis, and hitch-hiking.

We assessed the ability of the clinical staff to perform SCD-AMPS test by presenting an educational session and a demonstration of the rapid test with a focus on four steps: i) checking rapid tests for defects, ii) loading blood, iii) centrifugation of tests, and iv) interpretation of rapid test results. The staff was able to handle the rapid test and ask questions about the protocol and interpretation. Afterwards, participants were surveyed to assess their comfort with each of the four steps. Their familiarity and comfort with common lateral flow rapid tests was also assessed as a benchmark for usability. Participants rated tests using a five-point scale with 1 being “very difficult to use”, 3 being “okay to use”, and 5 being “very easy to use.” The clinical staff rated the ease of use of lateral flow tests, such a rapid diagnostic test for malaria, as 3.4.

Each step was rated for ease on the same 5 point scale. The initial set up was given an average rating of 3.3. The loading step was given an average rating of 3.4. Running the tests was given an average rating of 3.3. Reading results was given an average rating of 3.3. Every step was, thus, comparable to the ease of use of existing rapid tests.

## Conclusions

A density-based diagnostic for sickle cell disease using AMPSs has the potential to fill a critical healthcare gap in low-resource settings. Although sensitivity and specificity are not as high as those of gold-standard methods like high performance liquid chromatography and iso-electric focusing, these tests could provide actionable information when coupled with patient history and clinical presentation. Many clinics, such as those visited in Zambia, do not have the infrastructure to support the equipment needed for gold-standard methods but could run SCD-AMPS tests. Solubility tests, such as Sickledex (Streck), are often used to screen for SCD in clinics in India and sub-Saharan Africa, but they cannot differentiate between SCD and the largely benign, sickle cell trait. Although SCD-AMPS do not distinguish Hb AA from sickle cell trait, SCD-AMPS do discriminate between SCD and sickle cell trait.

The diagnostic ability of the SCD-AMPS depends on the presence of dense, sickled cells in the blood. These cells may not be present in newborns, and hence SCD-AMPS may not be appropriate for neonatal screening. Testing of SCD-AMPS on children under six months old is required to ascertain the minimum age at which the test will be useful for screening. After six months, however, the fraction of dense cells present is sufficient to allow use of SCD-AMPS-2. Coupled with vaccination programs, screening children for SCD with an SCD-AMPS test could provide useful information on prevalence.

SCD-AMPS are also acceptably simple to use; Initial demonstrations with staff at rural clinics in Zambia indicate that SCD-AMPS tests are comparable to standard rapid diagnostic tests for malaria in ease-of-use. Although the need for centrifugation and a battery may make the test impractical for a community health worker visiting households, we demonstrated that the equipment could be used in rural health centers with access to solar power. The capability to diagnose sickle cell disease at primary health centers and rural health centers in places like Zambia would allow for the targeted use of appropriate interventions (e.g., vaccines, prophylactic penicillin, and supplements for folate and iron) [24], [26], [27]. These interventions could reduce child mortality and improve the quality of life for those that live with undiagnosed sickle cell disease.

## Materials and Methods

### Chemicals

We used the following polymers: poly(ethylene glycol) (Sigma-Aldrich; MW  = 20000 Da), Ficoll (Sigma-Aldrich; MW  = 70000 Da and 400000 Da), dextran (Spectrum Chemical; 500000 Da), and poly(vinyl alcohol) (PVA) (Polysciences; MW  = 3000 Da)—formed by hydrolyzing 75% of poly(vinyl acetate). Solutions of AMPSs contained the following chemicals: ethylenediaminetetraacetic acid disodium salt (EDTA) (Sigma-Aldrich), potassium phosphate monobasic (EMD), sodium phosphate dibasic (Mallinkrodt AR), sodium chloride (EMD), MgCl_2_ (USB), and Nycodenz (Axis-Shield PoC).

### Components

We purchased the following components to assemble our rapid tests: heparinized, polycarbonate microhematocrit tubes (Iris Sample Processing), vinyl putty (Critoseal, Leica), silicone rubber tubing with an inner diameter of 1.02 mm and an outer diameter of 2.06 mm (Helix Mark, Helix Medical), glue (Krazy Glue), rubber caps (Critocaps, Leica), foil-lined pouches (Vapor-Flex VF48, LPS Industries), and shipping labels (5163, Avery). Templates to punch holes in capillary tubes and meter the volume of polymer to fill were printed in acrylonitrile butadiene styrene (ABS) using a 3D printer (Fortus 400mc, Dimension).

Blood collection used vacutainers (Becton Dickinson) coated with EDTA for 2 mL. Aliquots of the collected blood were transferred to vacutainers with no coating (Becton Dickinson).

### Standard Blood Tests

CBCs were performed at UTH using a Sysmex XT 2000i hematology analyzer. Calibrations and quality controls were performed daily. HE was performed at UTH using a semi-automated SAS1/2 from Helena Biosciences, LLC. Gels and hemoglobin controls were purchased from Helena Biosciences, LLC through their Sonergy Diagnostics, the local vendor in Zambia. Controls were run with every gel to ensure proper calibration and performance of the gels. For quantitative analysis of hemoglobin, we scanned gels using a transmission scanner (Epson Perfection V550) and analyzed the image using Platinum 4.1 software (Helena Biosciences).

### Classification of Subjects Based on Results from Hemoglobin Electrophoresis

Most subjects were easily classified based on the results from hemoglobin electrophoresis. No Hb C was detected in any of the subjects. Subjects with no detectable Hb S were classified as HbAA. Subjects with Hb A>50% and Hb S <50% were classified as Hb AS. Patients with no detectable Hb A and with detectable Hb S were classified as Hb SS. Hb F, when present, was quantified for all subjects. Of the over 500 subjects tested, we were then left with 12 subjects that had Hb S>50% but also had detectable Hb A. We classified these subjects as positive for SCD for the purposes of the study. All had either elevated reticulocyte counts or low hemoglobin concentrations. Their Hb S concentrations ranged from 54–78%. Based on the CBC results from these subjects, three of the subjects had a microcytic and hypochromic anemia. These 12 subjects may be SCD patients with Hb Sβ+ or they may have been transfused. In this study, these subjects were classified as having SCD.

### Statistics

Statistical analysis was done using R (http://www.r-project.org). We used Bayesian confidence intervals (Jeffreys prior) for the binary data that was used to make point estimates of sensitivity and specificity. We used a two-sided version of Fisher's exact test to test for significant differences between performances of the SCD-AMPS on different categorical data (e.g., recent crisis, different batches of SCD-AMPS, samples stored at different time intervals).

### Human Subjects Research

We obtained written consent from all participants and/or their legal guardians for the study of SCD-AMPS at UTH and from participants from the Harvard team who provided samples for demonstrations. The consenting process, protocol, and all associated materials were approved by the Institutional Review Board (IRB) of the Faculty of Arts and Sciences at Harvard University, the Committee on the Use of Human Subjects (CUHS) (Federal Wide Assurance (FWA) # 00004837, IRB Identification # 00000109) under the reference numbers 23799 and 16779. The process, protocol, and materials were also approved by a local Institutional Review Board in Lusaka, Zambia, the ERES Converge Committee (FWA # 00011697, IRB Identification # 00005948), under the reference number 2013-Jan-013. Verbal consent was obtained for participants of the survey of health workers in rural health centers in Zambia. The survey instrument did not include any identifying information, nor collected any information deemed potentially private by either CUHS or ERES Converge. An approved script was used to explain the survey and included a checkbox to indicate whether consent was provided. This survey of rural health workers in Zambia and the oral consent process was deemed exempt of need for full IRB review by CUHS (Ref. No. IRB13-1519) and underwent full IRB review and approval by ERES Converge (Ref. No. 2013-Sept-007).

### Supporting Information

Additional details regarding the fabrication, testing, and evaluation of the SCD-AMPS tests and the samples are provided in **S1 Text**.

## Supporting Information

S1 Figure **Schematic of the fabrication of SCD-AMPS tests.** Plastic microhematocrit tubes (A) insert into a holder (B) and can then be punctured with a pushpin (C) and metered with a marker (D). After blowing out debris with an airgun, we add a silicone sleeve to cover the hole in the side of the tube (E). We then added a well-mixed AMPS solution (F) and seal the bottom of the tube with putty (G). After a quick spin (H), the initial metering mark is removed (I) and replaced with a line to mark the level of the volume of the test (J). We then seal the bottom of the test with glue (K) and cover the open end with a rubber cap (L). A dozen completed tests fit into a foil lined pouch (M). We add 4 mL of water (N) and seal the package (O).
(TIF)

S2 Figure **Process to perform a rapid test for SCD with SCD-AMPS.** The user opens a packet and pour out the water (A) to retrieve the rapid tests (B). She then removes the rubber cap and centrifuges the test for 2 minutes (C). After checking that the phases have formed and the proper volume of liquid is present (D), the user slides down the silicone sleeve to reveal the punched hole (E) and wicks blood into the test until it reaches the hole (F). The user then slides the sleeve back over the hole (G) and centrifuges the test for 10 minutes (H). The test can then be read by eye (I).
(TIF)

S3 Figure **Flow diagram of recruitment and testing for the study to evaluate SCD-AMPS-2 and SCD-AMPS-3.** The reference test was hemoglobin (Hb) electrophoresis. SCD-AMPS-2 tests were classified as positive if a full layer of red or more was present at the bottom of the tube. SCD-AMPS-3 was classified as positive if there was a detectable red color at the bottom of the tube, including less than half a layer of red.
(TIF)

S4 Figure **The performance of the rapid test varied between batches shipped to Zambia.** The diagnostic accuracy was calculated for each of the five batches of rapid tests evaluated in Zambia show significant variation for both SCD-AMPS-2 (white) and SCD-AMPS-3 (light gray). Both the combined results (All) and individual batch results (1–5) are shown. Batch 1 showed the good discriminative ability with a diagnostic accuracy over 0.8 (80%) for both tests. Error bars indicate 95% confidence intervals. The number of subjects in each batch are listed in parentheses below the batch number.
(TIF)

S5 Figure **Effect of anticoagulants on the performance of SCD-AMPS.** Digital analysis of the red intensity at the bottom of the SCD-AMPS tests for a normal subject whose blood was treated with different concentrations of anticoagulant (EDTA). Samples were run in standard SCD-AMPS tubes treated with heparin (treated capillary) as well as SCD-AMPS loaded in tubes without any coating (untreated capillary). In all cases, the samples collected in the standard concentration of EDTA (5 mM) showed the least amount of red at the bottom of the tube. Variability in the concentration of anticoagulant during blood collection could be a source of false positives. Error bars represent standard error of the mean of triplicate experiments.
(TIF)

S1 Table **Time cutoffs for testing samples in the study.**
(DOCX)

S2 Table **Characterization of each batch of SCD-AMPS used in the study.**
(DOCX)

S3 Table **Specificity of SCD-AMPS on HbAA and HbAS (negative samples).**
(DOCX)

S4 Table **Assets at Rural Health Centers to treat SCD.**
(DOCX)

S1 Text **Supporting Information for Evaluation of a Density-based Rapid Diagnostic Test for Sickle Cell Disease in a Clinical Setting in Zambia.**
(DOCX)
