# Supplementary figures and images for: Evaluation of a Density-Based Rapid Diagnostic Test for Sickle Cell Disease in a Clinical Setting in Zambia
Source: PLoS One. 2014 Dec 9;9(12):e114540. doi: 10.1371/journal.pone.0114540 (PMC4260838; doi:10.1371/journal.pone.0114540)

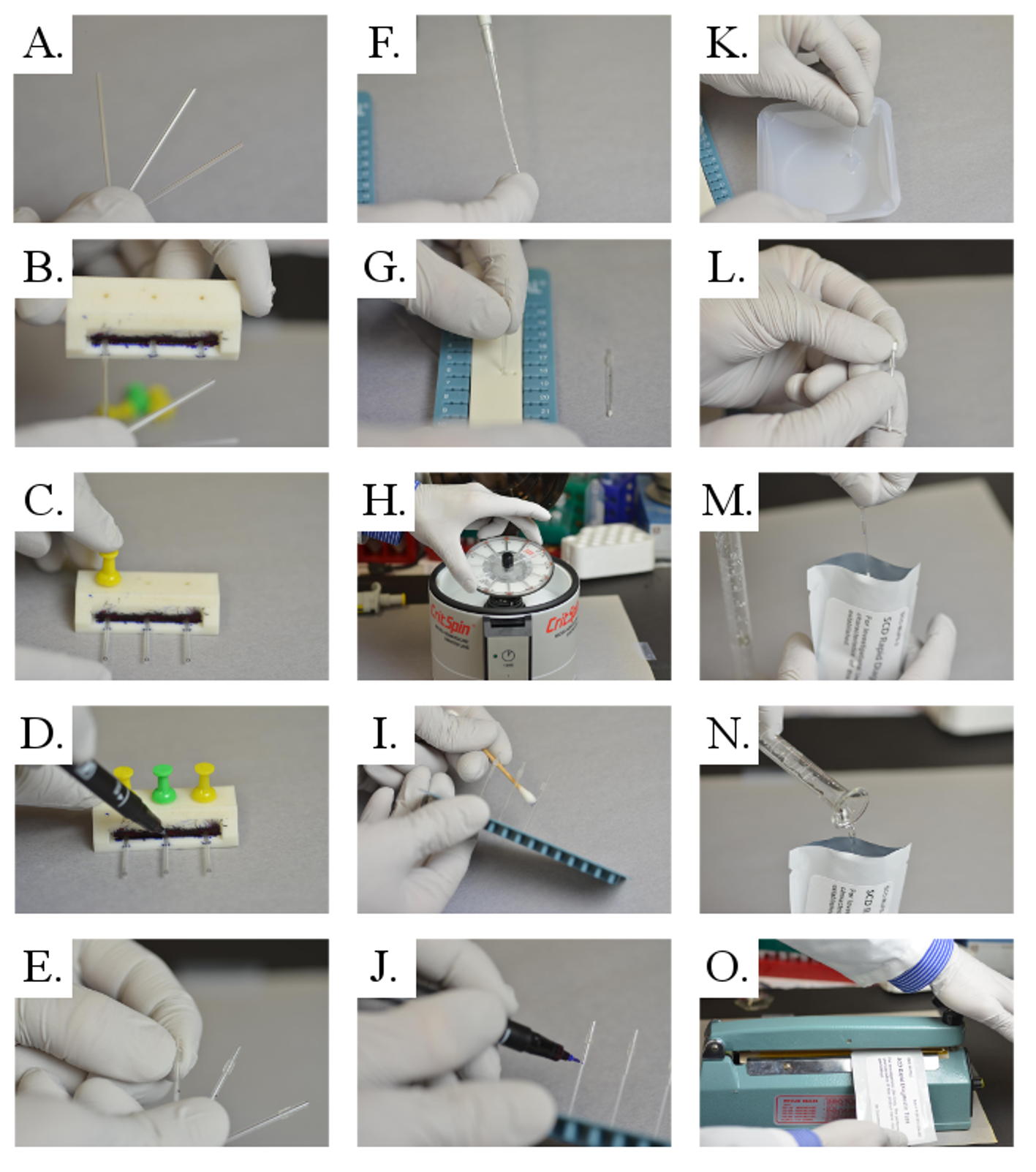

Supplement: S1 Figure — Schematic of the fabrication of SCD-AMPS tests. Plastic microhematocrit tubes (A) insert into a holder (B) and can then be punctured with a pushpin (C) and metered with a marker (D). After blowing out debris with an airgun, we add a silicone sleeve to cover the hole in the side of the tube (E). We then added a well-mixed AMPS solution (F) and seal the bottom of the tube with putty (G). After a quick spin (H), the initial metering mark is removed (I) and replaced with a line to mark the level of the volume of the test (J). We then seal the bottom of the test with glue (K) and cover the open end with a rubber cap (L). A dozen completed tests fit into a foil lined pouch (M). We add 4 mL of water (N) and seal the package (O). (TIF) [file pone.0114540.s001.tif]

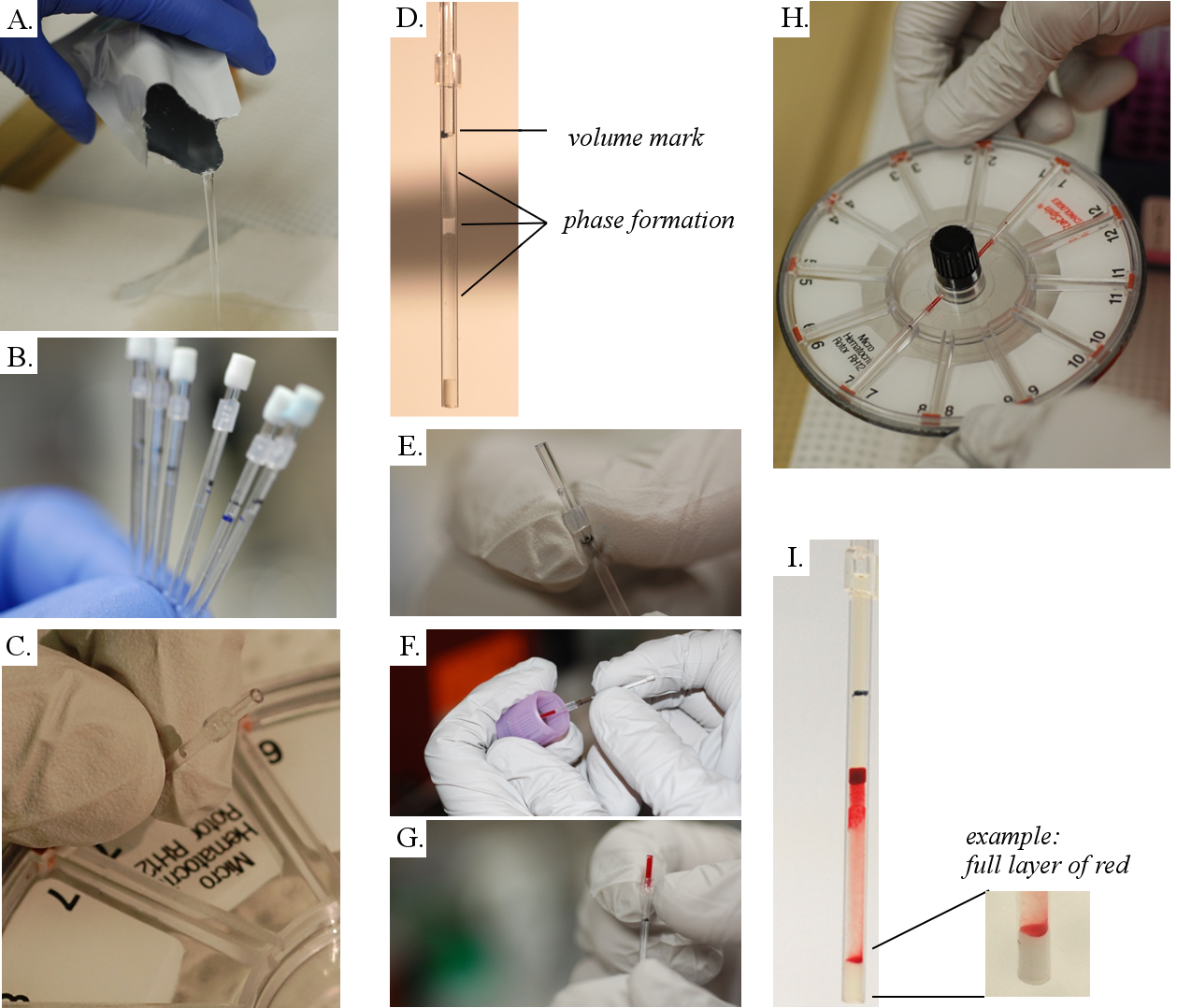

Supplement: S2 Figure — Process to perform a rapid test for SCD with SCD-AMPS. The user opens a packet and pour out the water (A) to retrieve the rapid tests (B). She then removes the rubber cap and centrifuges the test for 2 minutes (C). After checking that the phases have formed and the proper volume of liquid is present (D), the user slides down the silicone sleeve to reveal the punched hole (E) and wicks blood into the test until it reaches the hole (F). The user then slides the sleeve back over the hole (G) and centrifuges the test for 10 minutes (H). The test can then be read by eye (I). (TIF) [file pone.0114540.s002.tif]

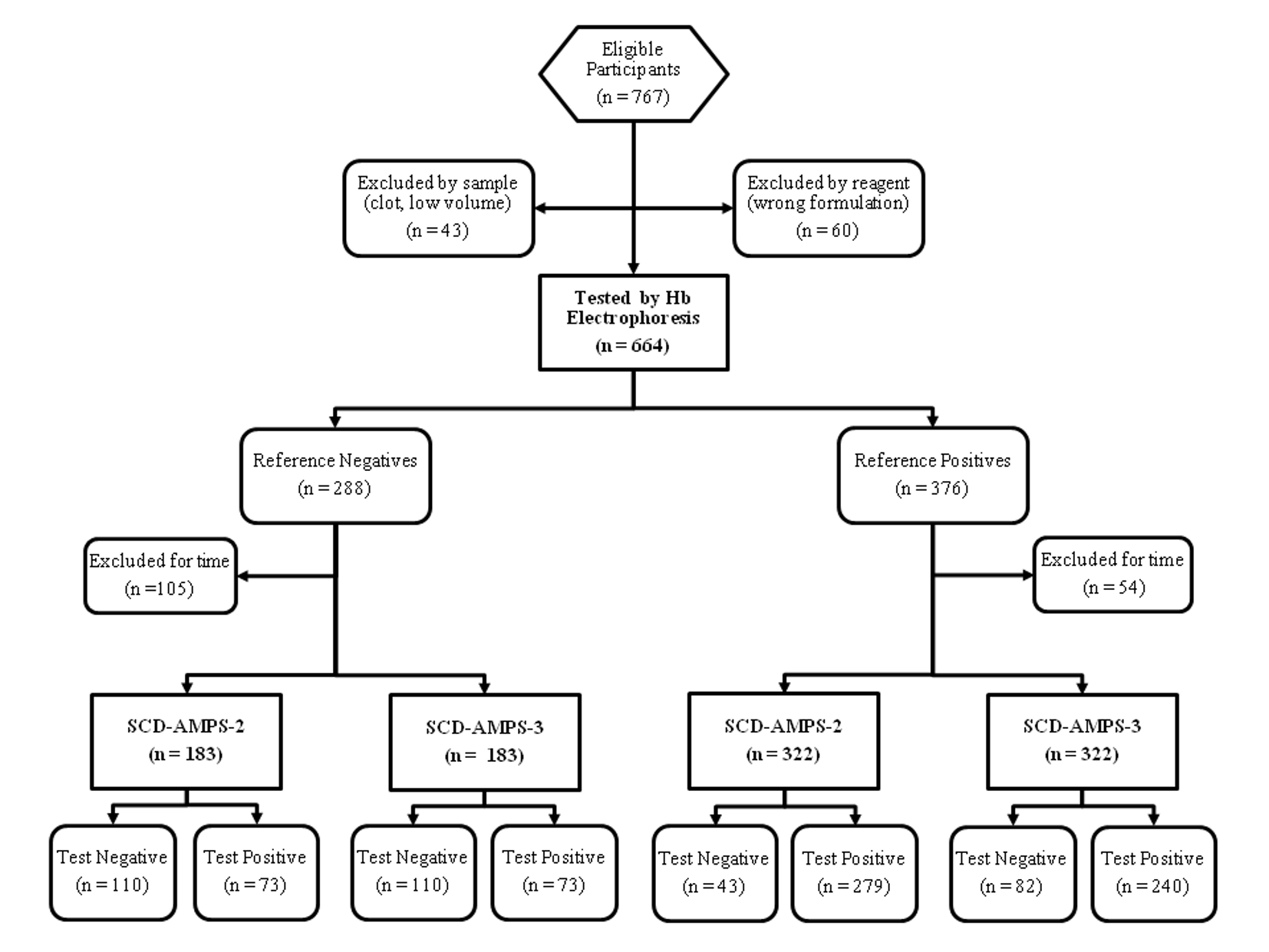

Supplement: S3 Figure — Flow diagram of recruitment and testing for the study to evaluate SCD-AMPS-2 and SCD-AMPS-3. The reference test was hemoglobin (Hb) electrophoresis. SCD-AMPS-2 tests were classified as positive if a full layer of red or more was present at the bottom of the tube. SCD-AMPS-3 was classified as positive if there was a detectable red color at the bottom of the tube, including less than half a layer of red. (TIF) [file pone.0114540.s003.tif]

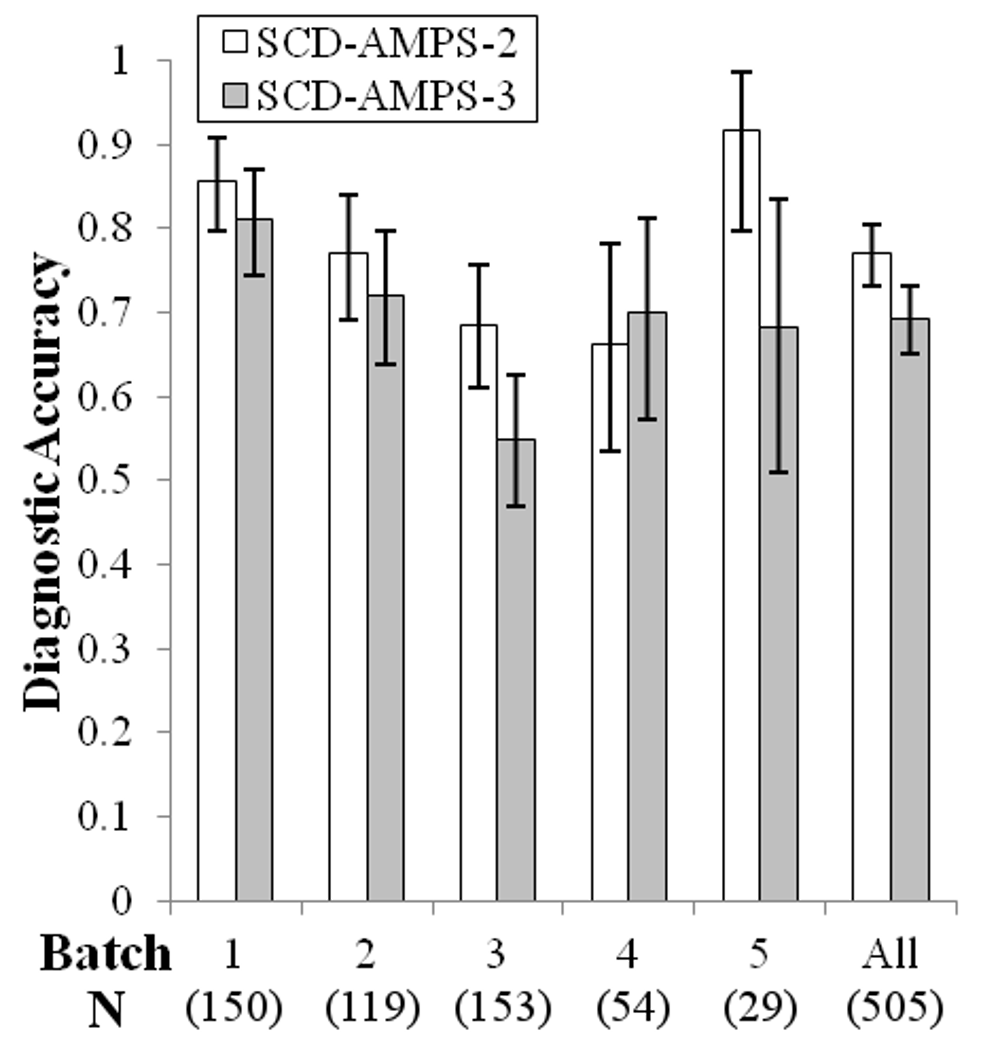

Supplement: S4 Figure — The performance of the rapid test varied between batches shipped to Zambia. The diagnostic accuracy was calculated for each of the five batches of rapid tests evaluated in Zambia show significant variation for both SCD-AMPS-2 (white) and SCD-AMPS-3 (light gray). Both the combined results (All) and individual batch results (1–5) are shown. Batch 1 showed the good discriminative ability with a diagnostic accuracy over 0.8 (80%) for both tests. Error bars indicate 95% confidence intervals. The number of subjects in each batch are listed in parentheses below the batch number. (TIF) [file pone.0114540.s004.tif]

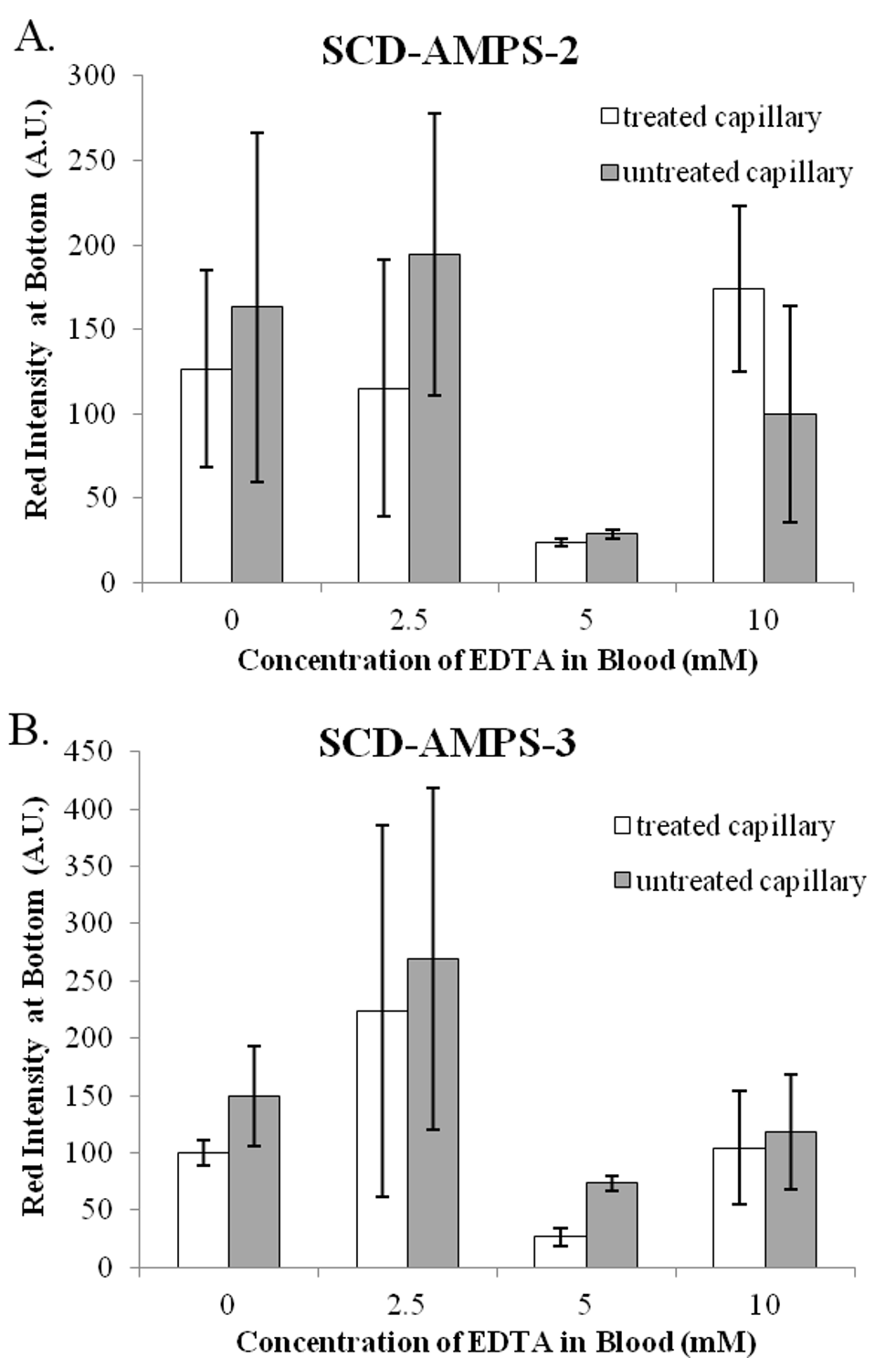

Supplement: S5 Figure — Effect of anticoagulants on the performance of SCD-AMPS. Digital analysis of the red intensity at the bottom of the SCD-AMPS tests for a normal subject whose blood was treated with different concentrations of anticoagulant (EDTA). Samples were run in standard SCD-AMPS tubes treated with heparin (treated capillary) as well as SCD-AMPS loaded in tubes without any coating (untreated capillary). In all cases, the samples collected in the standard concentration of EDTA (5 mM) showed the least amount of red at the bottom of the tube. Variability in the concentration of anticoagulant during blood collection could be a source of false positives. Error bars represent standard error of the mean of triplicate experiments. (TIF) [file pone.0114540.s005.tif]
