# Supplementary material for: Evaluation of a Density-Based Rapid Diagnostic Test for Sickle Cell Disease in a Clinical Setting in Zambia
Source: PLoS One. 2014 Dec 9;9(12):e114540. doi: 10.1371/journal.pone.0114540 (PMC4260838; doi:10.1371/journal.pone.0114540)
Supplement: S1 Table — Time cutoffs for testing samples in the study. (DOCX) [file pone.0114540.s006.docx]

**Table S1. Time cutoffs for testing samples in the study.**

| **Test Method** | **Maximum Time Before Testing** |
| --- | --- |
| SCD-AMPS-2; SCD-AMPS-3 | 48 hours |
| Hemoglobin Electrophoresis | 1 week |
| Complete Blood Count | 4 hours |
