# Supplementary material for: Evaluation of a Density-Based Rapid Diagnostic Test for Sickle Cell Disease in a Clinical Setting in Zambia
Source: PLoS One. 2014 Dec 9;9(12):e114540. doi: 10.1371/journal.pone.0114540 (PMC4260838; doi:10.1371/journal.pone.0114540)
Supplement: S2 Table — Characterization of each batch of SCD-AMPS used in the study. (DOCX) [file pone.0114540.s007.docx]

**Table S2**. **Characterization of each batch of SCD-AMPS used in the study.**

|  |  | **Top Phase** | | |  | **Bottom Phase** | | |  |  |
| --- | --- | --- | --- | --- | --- | --- | --- | --- | --- | --- |
| **Batch** | **SCD-AMPS** | **Density (g/cm^3^)** | **Osmolality (mOsm/kg)** | **pH** |  | **Density**  **(g/cm^3^)** | **Osmolality (mOsm/kg)** | **pH** | **Diagnostic Accuracy** | **95% CI** |
| 1 | 2 | 1.0776 | 292 | 7.37 |  | 1.1287 | 295 | 7.38 | 86% | (80,91) |
| 1 | 3 | 1.0754 | 293 | 7.36 |  | 1.1184 | 305 | 7.39 | 81% | (75,87) |
| 2 | 2 | 1.0790 | 293 | 7.35 |  | 1.1310 | 305 | 7.35 | 77% | (69,84) |
| 2 | 3 | 1.0787 | 297 | 7.44 |  | 1.1213 | 304 | 7.49 | 72% | (64,80) |
| 3 | 2 | 1.0782 | 299 | 7.39 |  | 1.1303 | NA | NA | 69% | (61,76) |
| 3 | 3 | 1.0776 | 301 | 7.39 |  | 1.1208 | NA | NA | 55% | (47,62) |
| 4 | 2 | 1.0788 | 306 | 7.40 |  | 1.1306 | 303 | NA | 66% | (53,78) |
| 4 | 3 | 1.0788 | 294 | 7.39 |  | 1.1204 | 297 | NA | 70% | (51,83) |
| 5 | 2 | 1.0786 | 303 | 7.40 |  | 1.1291 | 305 | NA | 92% | (80,99) |
| 5 | 3 | 1.0771 | 301 | 7.40 |  | 1.1184 | 304 | NA | 68% | (51,83) |
