# Supplementary material for: Evaluation of a Density-Based Rapid Diagnostic Test for Sickle Cell Disease in a Clinical Setting in Zambia
Source: PLoS One. 2014 Dec 9;9(12):e114540. doi: 10.1371/journal.pone.0114540 (PMC4260838; doi:10.1371/journal.pone.0114540)
Supplement: S3 Table — Specificity of SCD-AMPS on HbAA and HbAS (negative samples). (DOCX) [file pone.0114540.s008.docx]

**Table S3.** **Specificity of SCD-AMPS on HbAA and HbAS (negative samples).**

| **Genotype** | **Specificity** | **C.I.** |
| --- | --- | --- |
| *SCD-AMPS-2* | |  |
| HbAA | 60% | (52,68) |
| HbAS | 60% | (45,72) |
| *SCD-AMPS-3* | |  |
| HbAA | 58% | (50,66) |
| HbAS | 66% | (52,78) |
