# Supplementary material for: Evaluation of a Density-Based Rapid Diagnostic Test for Sickle Cell Disease in a Clinical Setting in Zambia
Source: PLoS One. 2014 Dec 9;9(12):e114540. doi: 10.1371/journal.pone.0114540 (PMC4260838; doi:10.1371/journal.pone.0114540)
Supplement: S4 Table — Assets at Rural Health Centers to treat SCD. (DOCX) [file pone.0114540.s009.docx]

Table S4. Assets at Rural Health Centers to treat SCD.

| **Interventions for SCD** | **Luena** | **Ipusukilo** |
| --- | --- | --- |
| Ward with beds | ✓ | ✓ |
| Non-opiate pain killers | ✓ | ✓ |
| Opiates |  |  |
| Iron supplements | ✓ | ✓ |
| Folic acid supplements | ✓ | ✓ |
| Antibiotics | ✓ | ✓ |
| Antimalarials (primaquine) | ✓ | ✓ |
| Pneumococcal vaccine | ✓ | ✓ |
| IV fluids | ✓ | ✓ |
| Transfusions |  |  |
