# Supplementary material for: Evaluation of a Density-Based Rapid Diagnostic Test for Sickle Cell Disease in a Clinical Setting in Zambia
Source: PLoS One. 2014 Dec 9;9(12):e114540. doi: 10.1371/journal.pone.0114540 (PMC4260838; doi:10.1371/journal.pone.0114540)
Supplement: S1 Text — Supporting Information for Evaluation of a Density-based Rapid Diagnostic Test for Sickle Cell Disease in a Clinical Setting in Zambia. (DOCX) [file pone.0114540.s010.docx]

**Text S1. Supporting Information for Evaluation of a Density-based Rapid Diagnostic Test for Sickle Cell Disease in a Clinical Setting in Zambia**

**Experimental Details**

**Fabrication. Figure S1** outlines the fabrication of a single test. We puncture a hole in the side of a polycarbonate capillary tube at a prescribed height using a customized holder and push-pin (**Figure S1**).

A silicone sleeve slides over the tube to open or close the hole. Using a pipette, we load a pre-mixed solution of an SCD-AMPS solution into the tube from one end and then seal that end with white, vinyl-based polymer sealant (Critoseal, Leica).

To ensure the vinyl sealant does not fail during shipping or storage, we dipped the sealed end of the tube in Krazy Glue and allowed the glue to set. After two minutes of centrifugation at 13,700 *g,* the phases of the SCD-AMPS system separated. We used a marker to indicate the highest level of the liquid in the tube at the time of fabrication as a quality control measure that could be checked before use. To reversibly seal the open end of the capillary, we used white rubber capillary covers (Critocaps, Leica).

AMPSs were made by weighing out the specified weight of polymer and Nycodenz into a volumetric flask. In this volume, we added a total concentration of 5 mM of EDTA, 2.96 mM of KH_2_PO_4_ and 9.36 mM of Na_2_HPO_4_. We added de-ionized water (MilliQ) to dissolve the solutes and bring the solution to the final volume. We then transferred the solutions to bottles and adjusted the pH using small volumes (less than 0.5% of the total volume) of concentrated NaOH and HCl to a final pH of 7.40 ± 0.02 (Orion 2 Star pH meter, Thermo Scientific). We added solid NaCl to the solution to adjust the osmolality to a 295 ± 15 mOsm/kg using a vapor pressure osmometer (Vapro 5600, Wescor). Solutions of AMPSs were stored in sealed bottles at 4 °C until the day of use to create rapid tests. We used a U-tube oscillator to measure density (DMA 35A, Anton Paar). All parameters (density, osmolality, and pH) were measured and tested and adjusted to the target ranges before adding the solution to the rapid tests.

**Evaluation of Fabrication Variability.** Metering a precise volume of blood into the rapid tests is potentially important to create reproducible results. In order to evaluate the effectiveness of our hole in the side of the tube, we scanned (V550, Epson) 48 tubes and used ImageJ software to evaluate the distance between the top of the tube and the bottom of the hole that was punched. The coefficient of variance (standard deviation/mean) of the distance was 2%. We then wicked blood into each tube and measured the distance between the top and bottom of the column of blood using a digital scanner. The coefficient of variance of the volume of blood that was loaded was 4%.

**Storage Tests.** We tested various methods to seal and package the rapid tests. For a method to seal the open end of the capillary we tried Parafilm, tape, various wax seals, and rubber caps (CritoCaps, Leica). Tubes were filled with water and sealed on one end with white putty (CritoSeal, Leica). The other end was then sealed with one of the above-mentioned methods. The mass and volume of the tests were measured and then the tests were put into an oven at 50 °C. Each day for one week, the tests were removed and measured again. At the end of the week, the tubes with the rubber caps had the least loss of volume and were the easiest to remove. We noted moisture and evaporation on the end of the tube that had been sealed with putty. Coating this seal with glue (Krazy Glue) minimized evaporation from this end.

We tested several packaging methods to store the sealed rapid tests. Using an impulse sealer (PFS-200), we sealed tests in plastic pouches modified from freezer bags (ZipLoc), foil-lined modified from food packaging (Lays), and foil-lined pouches from a vendor (LPS Industries). After adding rapid tests to each pouch and sealing them, we weighed the packages and added them to an oven at 50 °C. The pouches were weighed every day for one week. Only the foil lined pouches showed no measureable loss of mass. We chose the pouches from LPS Industries because of cost and availability. Opening the pouches revealed that even though water had not escaped the packaging, it had come out of the rapid tests; a small drop of water was generally found inside the pouch near the tubes. Based on the location, we found that either the glued end had broken or the caps had come off, potentially because of the build-up of pressure.

By adding water to the packaging along with the rapid tests, we created a moist environment. Performing similar stability tests as before revealed no measureable loss of volume in each of the individual rapid tests stored in the packaging with water. For packages containing 12 rapid tests, we added 4 mL of water.

In order to estimate the effects of long term storage on the SCD-AMPS tests, we packaged 300 rapid tests of SCD-AMPS-2 and SCD-AMPS-3 and stored them at 50 °C for one month. After this time, we let each package equilibrate to room temperature and then removed each rapid test. We removed the cap and used a razor blade to cut the putty seal off of the bottom of the test. Using a micropipette, we then removed the liquid from each of the tests and combined the samples of SCD-AMPS-2 and SCD-AMPS-3 in two separate conical tubes. The solutions were mixed with a vortex mixer and then centrifuged to separate the phases. Aliquots of the top and bottom phase of each system were removed and we measured density, osmolality, and pH. In each of the systems, the osmolality of the systems increased by ~10%. Density in each phase also increased by roughly 0.004 g/cm^3^. The pH of SCD-AMPS-2 increased from 7.40 to 7.56 while the pH of SCD-AMPS-3 was fairly stable, changing from 7.39 to 7.42. Some error may have been introduced into this method due to the difficulty in removing the entire polymer solution from the capillary. A method to assess the density in the rapid tests without removing the sample could provide a more accurate measure of density.

The observed increase in both osmolality and density may be the result of a loss of water during long term storage at high temperature. Also, some water may have evaporated inside the rapid test and coated the upper part of the capillary, leaving a more concentrated solution at the bottom of the tube that would have been removed. Centrifuging the tests before use to ensure that all the water has been added back to the solution could reduce this concentration. Increasing the length of the silicone sleeve could also reduce the potential for loss of volume in the tests. Additionally, the sodium heparin coating the capillary tubes may have increased the density and osmolality of the systems. Although these tests demonstrate the need for further improvement, the observed changes in density are less than the difference in density of normocytes and dense red blood cells present in SCD. With further improvements, SCD-AMPS could provide stable tests stored at room temperature for several months.

**Detailed Protocol.** All subjects were recruited from patients who were already indicated to have a venipuncture. During the clinically indicated venipuncture, an additional 2 mL of blood was collected in a vacutainer coated with EDTA and labeled with a study ID number. Nurses interviewed subjects and guardians to fill out a short survey to capture demographic data and patient history. These surveys were used to identify whether subjects should be excluded or included based on the recruitment criteria (**Table 1**).

Blood samples were stored in an insulated container with ice packs and transported to the reference laboratory running CBCs and HE. The laboratory technician then checked each sample to ensure it was properly labeled and to see if visible clots had formed. Samples with visible clots were noted and excluded from the study. The laboratory technician aliquoted samples of blood to untreated vacutainers labeled with the same study number and a study staff member then transported these samples to the pediatric laboratory where the rapid tests were run by a second laboratory technician. The laboratory technician running the rapid tests performed the procedure outlined in **Figure S2**, and made an initial reading using the evaluation levels depicted in **Figure 2**. During an initial training carried out in one day, the readers studied pictures of tubes with different levels of redness. During the pilot phase of the study, readers compared their readings with an expert reader. This training set of images was posted on the wall of the laboratory and available during all subsequent tests as a guide for the readers.

After the initial reading by the laboratory technician, a nurse from the study then performed an independent reading. In cases of conflicting readings, a second nurse read the rapid tests. All blood was tested within the amount of time specified by **Table S1** (i.e., the times specified by the manufacturer of the tests and equipment). Tests run after the times specified were marked as invalid and excluded from the analysis.

Each laboratory had a form to fill in values for each sample they received including the study number. The original questionnaire and these laboratory forms were collected at the end of each week and entered into a database using a user interface designed with Epi Info (CDC). The database was stored as an encrypted file and transferred to the Harvard team at the end of the study.

**Pilot Study**. For a pilot study, we recruited 20 participants fitting Subset 1, 11 participants fitting Subset 2, and six participants fitting Subset 4. Data from these participants were used to evaluate the recruitment and testing process and was analyzed separately from the main trial data.

**Main Study.** For the main study, we recruited a total of 767 eligible participants. Of these, a total of 505 subjects had complete sets of tests that were run under valid conditions to include in the study as discussed in the main text. The recruitment and testing of the samples are outlined in **Figure S3**.

**Evaluation of variability in reading tests between expert reader and trained readers.** One of the researchers who developed the SCD-AMPS at Harvard University trained a primary

reader and three secondary readers at UTH. After an initial one-day training, the readers at UTH shadowed the Harvard researcher in interpreting tests during the first days of the pilot phase of the study.

Halfway through the study, the researcher from Harvard returned and 100 rapid tests (51 SCD-AMPS-3 and 49 SCD-AMPS-2) were run on anonymous samples. The expert reader, as well as three of the UTH staff, independently read the results of each tests and compared responses. The three UTH staff readings were identical to the expert reader on 82% of the SCD-AMPS-3 and 60% of the SCD-AMPS-2. The difference in reading was generally only one level of redness off (e.g., “full layer of red” vs. “half layer of red”). On average, the UTH readers read tests with slightly higher levels of redness than the readings by the expert reader. This bias could cause an increase in false positives or decrease false negatives compared to results previously obtained for the SCD-AMPS test [1].

In addition to the variability introduced by the reader, some degree of variability in the sealing of the capillaries could lead to false negatives. The vinyl sealant retains the angle at which the tube was pressed into the sealant. During manufacturing, we held tubes vertically when sealing them. A slight angle, however, could result in an angled surface. This angled surface would require a larger volume of red blood cells to completely cover the white seal because it would have a larger surface area and depth that would need to be filled. Standardizing manufacturing procedures to eliminate deviation from horizontal in the surface of the sealant inside the capillaries may reduce the false negative rate.

**Variability in Performance by Batch**. During the six months of the study, a total of 5 batches each of SCD-AMPS-2 and SCD-AMPS-3 rapid tests were manufactured at Harvard University and shipped to UTH on ice. Batches generally arrived at UTH five days after shipping from Harvard. Batch 3 took approximately five days longer (a total of 10 days) to arrive at UTH after being shipped because a fire in the Nairobi airport disrupted international shipping routes. The conditions under which Batch 3 was stored while being held are unknown and, thus, we could not justify exclusion of data from this batch. The divergence in performance of Batch 3 from the other batches, however, does provide some insight into the role that shipping and storage could play in performance. When analyzing the performance of each test as a function of batch, we found large variations (**Figure 5**). Batch 1 showed best discriminative ability with diagnostic accuracies near or above 0.8 for both systems (n = 150). The density of the bottom phases of Batch 1 and 5 for both systems were ~0.002 g/cm^3^ less dense than they were for Batches 2-4. **Table S2** and **Figure S4** details the characteristics and performance of each batch.

**Evaluation of Potentially Confounding Factors.**

***Clotting:*** Blood samples in Zambia were collected in EDTA coated tubes. Variability in the total volume of blood drawn may have resulted in some samples receiving more or less than the recommended concentration of EDTA (~5 mM). To test what effect this variation might have, freshly drawn blood was treated with different concentrations of EDTA (0 mM, 2.5 mM, 5 mM, and 10 mM). Replicates (n = 3) of each of the treatments were loaded into SCD-AMPS-2 and SCD-AMPS-3 tests and centrifuged for 10 minutes. After centrifugation, we scanned each tube using a transmission scanner (Epson V550) and analyzed the intensity of the red at the bottom of the tube using a method previously described [1]. The signal from the blood treated with the standard concentration of EDTA (5 mM) was lowest (**Figure S5**); exposure of blood samples to either too much or too little EDTA could result in some false positives.

***Sickle Cell Trait:*** In solubility tests, people with sickle cell trait (Hb AS) are difficult to distinguish from those with SCD (Hb SS, Hb SC, and other variations). Interestingly, the specificity of SCD-AMPS-2 and SCD-AMPS-3 was similar between those with Hb AA and those with Hb AS; sickle cell trait is not a major source of false positives for the SCD-AMPS tests (**Table S3**). If improvements to the quality control of batches and anticoagulants used leads to improved performance, the ability to discriminate Hb AS from Hb SS could be a significant advantage for SCD-AMPS as way to screen for SCD.

**Assets in Rural Health Centers**. Sickle cell disease can be managed with the resources available in the two rural health centers that were visited in the Northern Province (**Table S4**).

**References**

1. Kumar AA, Patton MR, Hennek JW, Lee SYR, D’Alesio-Spina G, et al. (2014) Density-based Separation in Multiphase Systems Provides a Simple Method to Identify Sickle Cell Disease. Proc Natl Acad Sci U S A: doi:10.1073/pnas.1414739111. Available: http://www.pnas.org.ezp-prod1.hul.harvard.edu/content/early/2014/08/27/1414739111.short. Accessed 8 September 2014.
